# Autophagy and LAP in the Fight against Fungal Infections: Regulation and Therapeutics

**DOI:** 10.1155/2018/6195958

**Published:** 2018-03-06

**Authors:** Vasileios Oikonomou, Giorgia Renga, Antonella De Luca, Monica Borghi, Marilena Pariano, Matteo Puccetti, Giuseppe Paolicelli, Claudia Stincardini, Claudio Costantini, Andrea Bartoli, Teresa Zelante, Luigina Romani

**Affiliations:** ^1^Department of Experimental Medicine, University of Perugia, 06132 Perugia, Italy; ^2^Department of Pharmaceutical Science, University of Perugia, 06132 Perugia, Italy

## Abstract

Phagocytes fight fungi using canonical and noncanonical, also called LC3-associated phagocytosis (LAP), autophagy pathways. However, the outcomes of autophagy/LAP in shaping host immune responses appear to greatly vary depending on fungal species and cell types. By allowing efficient pathogen clearance and/or degradation of inflammatory mediators, autophagy proteins play a broad role in cellular and immune homeostasis during fungal infections. Indeed, defects in autophagic machinery have been linked with aberrant host defense and inflammatory states. Thus, understanding the molecular mechanisms underlying the relationship between the different forms of autophagy may offer a way to identify drugable molecular signatures discriminating between selective recognition of cargo and host protection. In this regard, IFN-*γ* and anakinra are teaching examples of successful antifungal agents that target the autophagy machinery. This article provides an overview of the role of autophagy/LAP in response to fungi and in their infections, regulation, and therapeutic exploitation.

## 1. Canonical and Noncanonical Autophagy

There is a growing appreciation for the complex role of autophagy proteins in immunity and inflammation. Autophagy plays a direct role in host defense by promoting the elimination of pathogens and indirectly by tight regulation of the innate and adaptive immune signaling pathways [1–4]. The canonical autophagy pathway (simply called “autophagy”) is a physiological cellular degradation process through which intracellular materials undergo lysosome-mediated self-degradation and recycling. It is activated in certain stressful conditions/situations such as starvation, hypoxia, or pathogen infection in order to preserve cellular homeostasis [5]. The process of autophagy is regulated by a large number of proteins that are also important in endosomal/phagosomal pathways, as well as by specific autophagy-related proteins (ATGs) [6]. A growing body of evidence suggests that ATG proteins have a broad role that goes beyond autophagy to include a broad impact on many aspects of human health and disease [7, 8]. In the fight against pathogenic microorganisms, the mechanism of autophagy/innate immune cross-talk has important effects on the induction and modulation of the inflammatory reaction during the infections [6, 9]. For instance, autophagy may temper inflammation by eliminating active inflammasomes via p62 ubiquitination [10]. Thus, defects in autophagy can worsen or directly contribute to aberrant host defense, inflammatory disease, and autoimmunity [7, 11].

In the last decade, an alternative form of autophagy has emerged, known as LC3- (microtubule-associated protein 1A/1B-light chain 3-) associated phagocytosis (LAP) or noncanonical autophagy. The connection between the autophagic machinery and phagocytosis can be viewed as a safe way to control and accelerate the lysosomal delivery of the phagosome and the degradation of its cargo (pathogens and engulfed cells). LAP is a unique pathway that engages cell-surface receptor signaling during phagocytosis via recruitment of a LC3-phosphatidylethanolamine (PE) conjugation system required for lysosomal fusion and maturation of the LAPosome [12]. Unlike canonical autophagy, the formation of the double-membrane autophagosome does not require the hierarchical intervention of all of the ATG proteins [13]. Rubicon, instead, is the master regulator of LAP [14]. Rubicon activates LAP when associated with the class III phosphatidylinositol (PI) 3-kinase [PI(3)K] complex containing a UV radiation resistance-associated gene (UVRAG) on the LAPosome—composed of a single membrane—and inhibits canonical autophagy by preventing Atg14L complex formation [14]. Moreover, Rubicon, by promoting phosphatidylinositol 3-phosphate [PI(3)P] localization and stabilization of the NOX2 NADPH oxidase complex to produce reactive oxygen species (ROS), facilitates the killing of ingested pathogens [14].

In addition to microbial defense, LAP has recently emerged as a major anti-inflammatory pathway with an important role in cellular homeostasis and physiology [15]. In particular, LAP prevented inflammation during dead cell clearance and protected against autoimmunity and inflammatory bowel disease [15]. Thus, understanding the molecular mechanisms underlying LAP's ability to modulate the inflammatory response during infection may have therapeutic implications. In this review, we discuss how canonical autophagy and LAP contribute to host defense against fungi and the possible therapeutic implications.

## 2. Canonical Autophagy and LAP in Host Defense against Fungal Pathogens

Most human fungal pathogens, such as *Candida* spp., *Cryptococcus neoformans*, and *Aspergillus fumigatus*, have not evolved as primary pathogens in healthy individuals but instead cause severe life-threatening diseases when the immune competence of the host is compromised [16, 17]. The innate immune mechanisms of the host phagocytes are the primary method of defense against fungal pathogens [17]. Phagocytic clearance of fungal pathogens begins with the activation of pathogen-associated molecular patterns—cell-surface fungal specific molecules—by pattern recognition receptors of phagocytes [16, 17] that triggers the induction of cytokines, chemokines, and other antimicrobial mediators to orchestrate inflammation and host defense [16].

**Figure 1:**
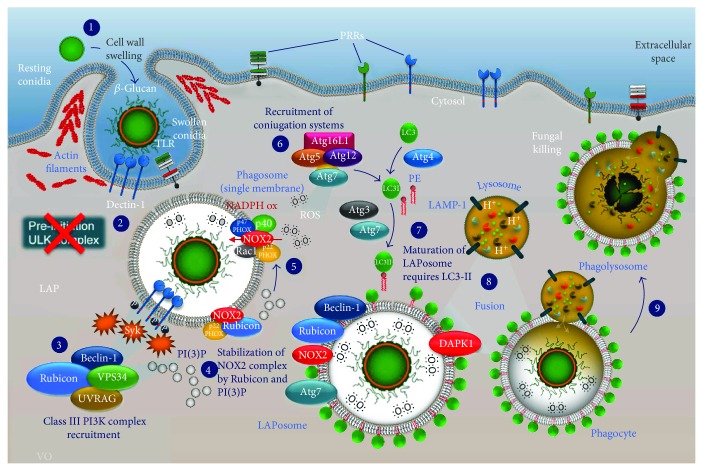
Overview of the LAP pathway during *Aspergillus fumigatus* infection. (1) Cell wall swelling of resting *Aspergillus* conidia (exposure of carbohydrate molecules, such as *β*-glucan) activates innate immune responses in host phagocytes. (2) Pattern recognition receptors (PPRs) on phagocytes (Dectin-1, TLRs) recognize *β*-glucan and internalize it through phagocytosis. During phagocytosis, swollen *Aspergillus* conidia trigger the Dectin-1/Syk kinase complex. (3) Subsequent activation of LAP occurs by recruitment of the class III PI3K complex (Rubicon/UVRAG/VPS34/Beclin-1) to the single-membrane phagosome. (4) The class III PI3K complex generates PI(3)P that localizes to phagosome with subsequent stabilization of the NOX2 complex by Rubicon and PI(3)P. (5) Complete assembly of the NOX2 NADPH oxidase complex is capable in optimal ROS production. (6) PI(3)P formation and ROS production lead to the recruitment of the downstream Atg5/Atg12/Atg16L1 conjugation complex, as well as of Atg7, Atg3, and Atg4, all of which are critical for the lipidation of LC3 (conjugation of LC3I to PE to form LC3II). (7) LAPosome maturation requires LC3II deposition and DAPK1 localization for anti-inflammatory activity. Finally, (8) fusion with LAMP-1-lysosome and (9) phagolysosome formation with fungal killing occur.

Several studies have demonstrated that LAP plays a critical role in antifungal immunity during fungal infection [14, 18–24] and is required for an efficient fungal killing [25]. LAP, but not canonical autophagy, participated in the degradation of engulfed *Aspergillus* conidia [14, 21, 22] (Figure 1). Dectin-1 played a pivotal role in regulating the induction of LAP. Dectin-1-deficient mice have impaired *β*-glucan recognition, resulting in increased susceptibility to fungal infections caused by *A. fumigatus* [26]. Moreover, genetic polymorphisms affecting human Dectin-1 have also been addressed as potential predictive factors that increase the susceptibility to invasive aspergillosis in immunocompromised patients [27]. Additional evidences for the involvement of LAP in the clearance of *A. fumigatus* were provided using LAP-deficient ATG7 mice that exhibited increased fungal burden, inflammation, and proinflammatory cytokine levels [28]. Kyrmizi et al. have recently reported that *β*-glucan surface exposure, upon germination of “swelling” *A. fumigatus* conidia, activates LAP via a Dectin-1/Src/Syk kinase signaling cascade and subsequent lipidate LC3 (LC3-II) recruitment to *Aspergillus*-containing phagosomes [19]. Recruitment of LC3 to *Aspergillus*-containing phagosomes was dependent on NADPH oxidase-mediated ROS production [19, 21]. Monocytes of patients with chronic granulomatous disease (CGD)—nicotinamide adenine dinucleotide phosphate (NADPH) oxidase-deficient—displayed defective recruitment of LC3 to phagosomes in response to internalized bacteria and *Aspergillus* [19–21]. In murine and human CGD, the inflammation and infectious susceptibility were regulated by LAP [20]. The loss of ROS production was associated with the reduced number of LC3-positive cells upon *A. fumigatus* infection and high levels of inflammasome/caspase activity, both functions being normalized by the treatment with anakinra [20]. Interestingly, treatment with corticosteroids inhibited Src/Syk signaling and ROS production, both of which resulted in impaired recruitment of LC3-II to phagosomes. This suggests that the inhibition of LAP by corticosteroids might contribute to the increased susceptibility that is associated with corticosteroid treatment [19]. These findings suggest that LAP is a drugable pathway in *Aspergillus* infections. Furthermore, Akoumianaki et al. have shown that cell wall melanin also blocks functional LAP against *A. fumigatus* [21]. Mechanistically, melanin inhibited NADPH oxidase-dependent activation of LAP by selectively excluding the p22phox subunit from the phagosome membrane [21]. Thus, melanin-induced LAP blockade is an important virulence strategy that confers resistance to killing by macrophages while promoting the development of invasive fungal infection [29]. Collectively, these studies confirmed that activation of LAP in response to *Aspergillus* conidia occurs through a Dectin-1/Syk kinase/NADPH-dependent mechanism. Martinez et al. also identified Rubicon as the molecular switch between repression of autophagy and the activation of LAP [14]. Specifically, they have shown the indispensable role of Rubicon in LAP of *A. fumigatus* and identified the autophagy proteins (Beclin-1, Atg7, UVRAG, VPS34 PI3-kinase complex I, and LC3-II) required for LAP [14]. The recruitment of Rubicon to LAPosomes was found to be dependent on the PI(3)K complex, and maturation of LAPosomes required ROS production through NOX2 [14]. LAP-deficient mice exhibited increased pathological inflammation, proinflammatory cytokines (such as IL-1*β*, IL-6, IL-12, and TNF-*α*), and fungal burden [14]. These data highlight both the molecular requirement of LAP and the central role of Rubicon in the immune response to *A. fumigatus*.

LAP also occurred in response to *Candida albicans* depending upon the exposure of enough *β*-glucan on the cell surface [30]. Previous studies have shown successful recruitment of LC3 to zymosan particles [14] and *β*-glucan-coated polystyrene beads [18, 30]. The recruitment of LC3 to *C. albicans*-containing phagosomes facilitated MHC class II presentation of fungal antigens in dendritic cells [18] and promoted fungicidal activity and expression of proinflammatory cytokines in macrophages [30]. Autophagy enhanced NF-*κ*B activity in response to the fungus through A20 sequestration, and this allowed the release of chemokines to recruit neutrophils. Accordingly, mice lacking autophagy in myeloid cells showed higher susceptibility to *C. albicans* infection due to the impairment in neutrophil recruitment [24]. However, the role of autophagy in vivo is not, as yet, clarified. Autophagy proteins ATG5 and ATG7 have been shown to play a protective role in murine [6, 15] but not in human systemic *C. albicans* infection [31]. It is likely that the role of autophagy proteins in host protection against *C. albicans* depends on the fungal species and may rely on specific autophagy components. Autophagy proteins are also involved in host response to *Cryptococcus neoformans*. ATG5, ATG9a, and ATG12 were engaged, but not required, in macrophages phagocytosing *C. neoformans*, and the proteins were recruited to the vicinity of vacuoles containing *C. neoformans* [23]. However, autophagy proteins and LC3 enhanced intracellular replication and escape of *C. neoformans* from vacuoles. Indeed, pharmacological inhibition of autophagy by 3-methyladenine and *Atg5* deficiency in myeloid cells [23] reduced the levels of *Cryptococcus* infection [32]. Further studies are needed to clarify the roles of autophagy and LAP in *Cryptococcus* infection.

## 3. DAPK1 Dampens Inflammation during Fungal LAP

**Figure 2:**
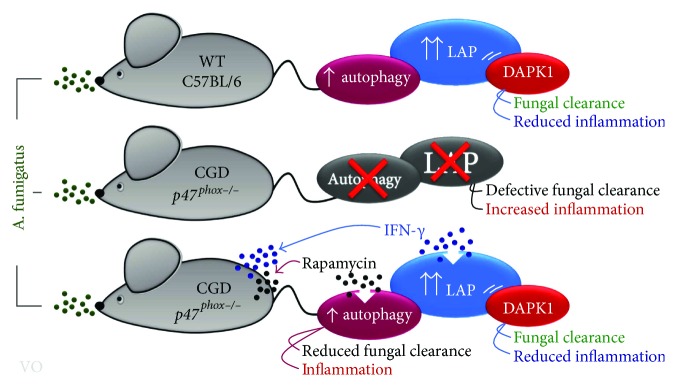
Proposed model of cross-regulation between LAP and canonical autophagy in *p47^phox−/−^* mice. The figure shows that both autophagy and LAP are defective in *p47^phox−/−^* mice. Rapamycin, a known inducer of canonical autophagy, failed to promote fungal clearance and reduce inflammation when administered to CGD mice as opposed to IFN-*γ* that promoted fungal clearance and reduced pathogenic inflammation by inducing the LAP/DAPK1 pathway. Thus, canonical autophagy and LAP are distinct pathways in *Aspergillus* infection, and LAP, more than canonical autophagy, is required for optimal antifungal resistance. CGD: chronic granulomatous disease; DAPK1: death-associated protein kinase 1; IFN-*γ*: interferon gamma; LAP: LC3-associated phagocytosis; WT: wild type.

The ability of LAP to execute pathogen clearance while simultaneously attenuating inflammation and autoimmunity [33] points to LAP as an ideal drugable pathway in immune homeostasis during infections and demands for a better understanding of the molecular mechanisms behind it. Putative mechanisms by which LAP may regulate immune function have recently been described [33]. We have recently identified a mechanism by which inflammation is regulated during LAP, that is, through death-associated protein kinase 1 (DAPK1) [22]. DAPK1 is a Ca^+2^/calmodulin-regulated serine/threonine kinase originally identified as the effector of IFN-*γ*-induced cell death, with a key role to suppress tumor growth [34]. DAPK1 is a molecule that participates in multiple signaling cascades [35] with pleiotropic effects on the regulation of inflammation depending on cell types and environmental cues [34]. In the lungs, DAPK1 suppressed lung inflammation and airway injury [36]. Of note, the expression of DAPK1 was regulated by the IFN-*γ* signaling pathway via the proteolytic cleavage and nuclear translocation of the endoplasmic reticulum stress-activated transcriptional factor ATF6 in association with the phosphorylated C/EBP-*β* transcription factor [37, 38]. It is of interest that IFN-*γ* plays a critical role in antifungal immunity [16] with multiple roles ranging from autophagy to negative regulation of the inflammatory response. Accordingly, IFN-*γ* has been implicated as a treatment in invasive fungal infections [16]. We found that DAPK1 turned out to play a dual protective role against *Aspergillus* infection. It was recruited to *Aspergillus*-containing phagosomes in a manner dependent on Rubicon and was essential for LAP and fungal clearance (Figures 1 and 2). In addition, DAPK1 was able to induce ubiquitination of the nucleotide-binding domain-like receptor protein 3 (NLRP3) inflammasome in an F-box leucine-rich repeat protein 2-dependent manner, thus restraining the inflammatory response. Consistent with the involvement of DAPK1 in LAP in response to the fungus, DAPK1 inhibition did not affect rapamycin-induced autophagy [22], a finding confirming that DAPK1 is not involved in starvation-induced autophagy [39]. Clinical translation of these results was evaluated in two different models. Given the dependence of DAPK1 on NOX2, we found that DAPK1 expression was lacking in a CGD mouse model and in monocytes from CGD patients and could be restored upon treatment with IFN-*γ*. In addition, in a cohort of hematopoietic stem cell-transplanted patients, we could show that genetic deficiency of DAPK1 was associated with increased inflammation and susceptibility to aspergillosis [22]. Thus, the IFN-*γ*/DAPK1 signaling pathway not only mediates LAP in response to *A. fumigatus* but also inhibits NLRP3 activation, restrains pathogenic inflammation, and is a drugable pathway.

## 4. Cross-Regulation between LAP and Canonical Autophagy in Fungal Infections

**Figure 3:**
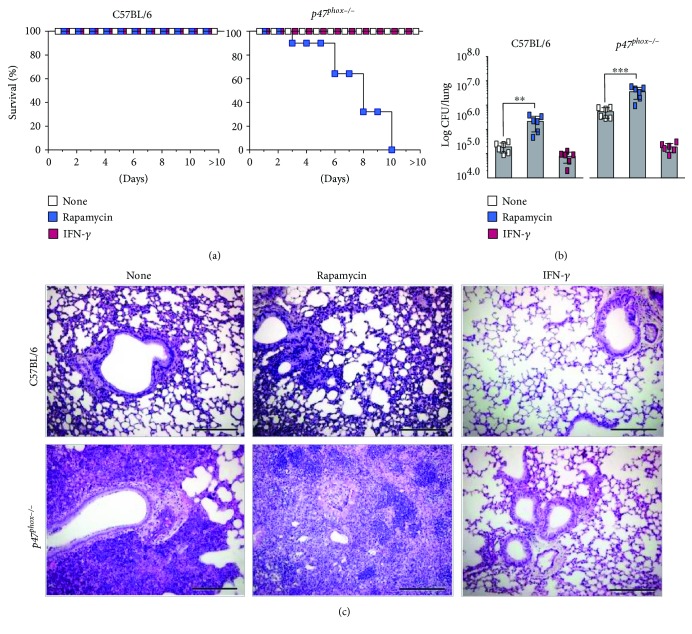
IFN-*γ* restores defective LAP in *p47^phox−/−^* mice. C57BL/6 and *p47^phox−/−^* mice (*n* = 6) were infected intranasally with *A. fumigatus* conidia and treated with rapamycin (10 mg/kg/ip for 3 consecutive days) and IFN-*γ* (20,000 U/mouse/ip for 7 consecutive days) for one week. Mice were sacrificed 7 days after infection and assessed for (a) survival (%), (b) fungal growth (log10 CFU ± SD), and (c) lung histology (PAS staining; scale bar represents 200 *μ*m). Data are pooled or representative (histology) of three independent experiments. ^∗∗^*P* < 0.001 and ^∗∗∗^*P* < 0.0001. Treated versus untreated (none) mice. One-way ANOVA, Bonferroni post test.

The above results clearly indicate that LAP pivotally contributes to the control of infection and inflammation at the fungus/host interface. However, the contribution of canonical autophagy in this process is less clear. In this regard, *ULK1*^−/−^ mice have been demonstrated to be as resistant to the infection as canonical autophagy-sufficient mice [14]. Consistent with this observation, we have shown that rapamycin, a known inducer of canonical autophagy [40], failed to increase resistance to infection when administered to conventional C57BL/6 mice with pulmonary aspergillosis [41]. This result would implicate a limited role, if any, of canonical autophagy in fungal clearance and inflammation. This appeared to be the case as treatment of CGD mice with rapamycin did not rescue the mice from infection. Actually, treatment with rapamycin reduced survival, impeded fungal clearance, and greatly promoted inflammation (Figures 3(a)–3(c)). As already shown [42], treatment with IFN-*γ* promoted fungal clearance and reduced pathogenic inflammation (Figures 3(a)–3(c)). Altogether, these results confirm the unique role of LAP in *Aspergillus* infection and suggest a possible cross-regulatory activity between canonical autophagy and LAP during infection (Figure 2). This would not come as a surprise given that Rubicon is known to inhibit canonical autophagy [14]. However, whether and how canonical autophagy interferes with the development of LAP is less clear. Given that IFN-*γ* could activate autophagy through tryptophan starvation via the indoleamine 2,3-dioxygenase 1 enzyme [43], we resorted to *Indo^−/−^* mice to assess the possible regulatory activity of canonical autophagy on LAP. We found that IFN-*γ*-induced autophagy was defective in *Indo^−/−^* macrophages and not modified by DAPK1 inhibition [22]. In contrast, LC3-dependent phagocytosis of the fungus was observed in *Indo^−/−^* macrophages in vitro, and DAPK1 expression increased in *Indo^−/−^* mice upon infection in vivo. Blocking DAPK1 greatly increased the fungal burden and dissemination in *Indo^−/−^* mice as well as the inflammatory cell recruitment in the lungs and in the bronchoalveolar lavage fluid [22], a finding indicating that the starvation-induced autophagy and LAP are distinct, yet complementary, pathways in *Aspergillus* infection.

## 5. Conclusions

The autophagy/LAP machinery is activated in response to fungi, thus making the pathway amenable for therapeutics. In this regard, IFN-*γ* and anakinra are teaching examples of successful antifungal agents that target the autophagy machinery. However, the outcomes of autophagy/LAP in shaping host immune responses appear to greatly vary depending on fungal species. It is likely that these outcomes also depend on the types of immune cells involved. Ultimately, one most intriguing aspect that needs further elucidation is the relationship between selective forms of autophagy and noncanonical autophagy in different cell compartments. Learning to distinguish between canonical and noncanonical autophagy may offer a way to identify molecular signatures of autophagy that are beyond the selective recognition of cargo to include host-protective signatures.

## Abbreviations

ATGs: Autophagy-related genes
ATF6: Activating transcription factor 6
C/EBP: CCAAT/enhancer-binding proteins
CGD: Chronic granulomatous disease
DAPK1: Death-associated protein kinase 1
IFN-*γ*: Interferon gamma
*Indo^−/−^* mice: Indoleamine 2,3-dioxygenase 1 enzyme-deficient mice
LAP: LC3-associated phagocytosis
LC3: Microtubule-associated protein 1A/1B-light chain 3
MHC: Major histocompatibility complex
NADPH: Nicotinamide adenine dinucleotide phosphate oxidase
NLRP3: Nucleotide-binding domain-like receptor protein 3
PE: Phosphatidylethanolamine
PI(3)P: Phosphatidylinositol 3-phosphate
PI(3)K: Phosphatidylinositol (PI) 3-kinases
ROS: Reactive oxygen species
Rubicon: Run domain Beclin-1-interacting and cysteine-rich domain-containing protein
Syk: Spleen tyrosine kinase
ULK: UNC-51-like autophagy-activating kinase 1
UVRAG: UV radiation resistance-associated gene.

## References

[1] Nedjic J., Aichinger M., Emmerich J., Mizushima N., Klein L. (2008). Autophagy in thymic epithelium shapes the T-cell repertoire and is essential for tolerance. *Nature*.

[2] Saitoh T., Akira S. (2010). Regulation of innate immune responses by autophagy-related proteins. *The Journal of Cell Biology*.

[3] Cadwell K. (2016). Crosstalk between autophagy and inflammatory signalling pathways: balancing defence and homeostasis. *Nature Reviews Immunology*.

[4] Deretic V., Saitoh T., Akira S. (2013). Autophagy in infection, inflammation and immunity. *Nature Reviews Immunology*.

[5] Qian M., Fang X., Wang X. (2017). Autophagy and inflammation. *Clinical and Translational Medicine*.

[6] Sun Q., Fan J., Billiar T. R., Scott M. J. (2017). Inflammasome and autophagy regulation: a two-way street. *Molecular Medicine*.

[7] Subramani S., Malhotra V. (2013). Non-autophagic roles of autophagy-related proteins. *EMBO Reports*.

[8] Shibutani S. T., Saitoh T., Nowag H., Munz C., Yoshimori T. (2015). Autophagy and autophagy-related proteins in the immune system. *Nature Immunology*.

[9] Vural A., Kehrl J. H. (2014). Autophagy in macrophages: impacting inflammation and bacterial infection. *Scientifica*.

[10] Shi C. S., Shenderov K., Huang N. N. (2012). Activation of autophagy by inflammatory signals limits IL-1*β* production by targeting ubiquitinated inflammasomes for destruction. *Nature Immunology*.

[11] Netea-Maier R. T., Plantinga T. S., van de Veerdonk F. L., Smit J. W., Netea M. G. (2016). Modulation of inflammation by autophagy: consequences for human disease. *Autophagy*.

[12] Mehta P., Henault J., Kolbeck R., Sanjuan M. A. (2014). Noncanonical autophagy: one small step for LC3, one giant leap for immunity. *Current Opinion in Immunology*.

[13] Codogno P., Meijer A. J. (2013). Autophagy in the liver. *Journal of Hepatology*.

[14] Martinez J., Malireddi R. K. S., Lu Q. (2015). Molecular characterization of LC3-associated phagocytosis reveals distinct roles for Rubicon, NOX2 and autophagy proteins. *Nature Cell Biology*.

[15] Martinez J., Cunha L. D., Park S. (2016). Noncanonical autophagy inhibits the autoinflammatory, lupus-like response to dying cells. *Nature*.

[16] Romani L. (2011). Immunity to fungal infections. *Nature Reviews Immunology*.

[17] Erwig L. P., Gow N. A. R. (2016). Interactions of fungal pathogens with phagocytes. *Nature Reviews Microbiology*.

[18] Ma J., Becker C., Lowell C. A., Underhill D. M. (2012). Dectin-1-triggered recruitment of light chain 3 protein to phagosomes facilitates major histocompatibility complex class II presentation of fungal-derived antigens. *The Journal of Biological Chemistry*.

[19] Kyrmizi I., Gresnigt M. S., Akoumianaki T. (2013). Corticosteroids block autophagy protein recruitment in *Aspergillus fumigatus* phagosomes via targeting dectin-1/Syk kinase signaling. *Journal of Immunology*.

[20] de Luca A., Smeekens S. P., Casagrande A. (2014). IL-1 receptor blockade restores autophagy and reduces inflammation in chronic granulomatous disease in mice and in humans. *Proceedings of the National Academy of Sciences of the United States of America*.

[21] Akoumianaki T., Kyrmizi I., Valsecchi I. (2016). *Aspergillus* cell wall melanin blocks LC3-associated phagocytosis to promote pathogenicity. *Cell Host & Microbe*.

[22] Oikonomou V., Moretti S., Renga G. (2016). Noncanonical fungal autophagy inhibits inflammation in response to IFN-*γ* via DAPK1. *Cell Host & Microbe*.

[23] Nicola A. M., Albuquerque P., Martinez L. R. (2012). Macrophage autophagy in immunity to *Cryptococcus neoformans* and *Candida albicans*. *Infection and Immunity*.

[24] Kanayama M., Inoue M., Danzaki K., Hammer G., He Y. W., Shinohara M. L. (2015). Autophagy enhances NF*κ*B activity in specific tissue macrophages by sequestering A20 to boost antifungal immunity. *Nature Communications*.

[25] Sprenkeler E. G. G., Gresnigt M. S., van de Veerdonk F. L. (2016). LC3-associated phagocytosis: a crucial mechanism for antifungal host defence against *Aspergillus fumigatus*. *Cellular Microbiology*.

[26] Werner J. L., Metz A. E., Horn D. (2009). Requisite role for the dectin-1 *β*-glucan receptor in pulmonary defense against *Aspergillus fumigatus*. *Journal of Immunology*.

[27] Cunha C., di Ianni M., Bozza S. (2010). Dectin-1 Y238X polymorphism associates with susceptibility to invasive aspergillosis in hematopoietic transplantation through impairment of both recipient- and donor-dependent mechanisms of antifungal immunity. *Blood*.

[28] Sanjuan M. A., Dillon C. P., Tait S. W. G. (2007). Toll-like receptor signalling in macrophages links the autophagy pathway to phagocytosis. *Nature*.

[29] Chamilos G., Akoumianaki T., Kyrmizi I., Brakhage A., Beauvais A., Latge J. P. (2016). Melanin targets LC3-associated phagocytosis (LAP): a novel pathogenetic mechanism in fungal disease. *Autophagy*.

[30] Tam J. M., Mansour M. K., Khan N. S. (2014). Dectin-1–dependent LC3 recruitment to phagosomes enhances fungicidal activity in macrophages. *The Journal of Infectious Diseases*.

[31] Smeekens S. P., Malireddi R. K., Plantinga T. S. (2014). Autophagy is redundant for the host defense against systemic *Candida albicans* infections. *European Journal of Clinical Microbiology & Infectious Diseases*.

[32] Qin Q. M., Luo J., Lin X. (2011). Functional analysis of host factors that mediate the intracellular lifestyle of *Cryptococcus neoformans*. *PLoS Pathogens*.

[33] Heckmann B. L., Boada-Romero E., Cunha L. D., Magne J., Green D. R. (2017). LC3-associated phagocytosis and inflammation. *Journal of Molecular Biology*.

[34] Lai M. Z., Chen R. H. (2014). Regulation of inflammation by DAPK. *Apoptosis*.

[35] Bialik S., Kimchi A. (2014). The DAP-kinase interactome. *Apoptosis*.

[36] Nakav S., Cohen S., Feigelson S. W. (2012). Tumor suppressor death-associated protein kinase attenuates inflammatory responses in the lung. *American Journal of Respiratory Cell and Molecular Biology*.

[37] Gade P., Ramachandran G., Maachani U. B. (2012). An IFN-*γ*–stimulated ATF6–C/EBP-*β*–signaling pathway critical for the expression of death associated protein kinase 1 and induction of autophagy. *Proceedings of the National Academy of Sciences of the United States of America*.

[38] Kalvakolanu D. V., Gade P. (2012). IFNG and autophagy: a critical role for the ER-stress mediator ATF6 in controlling bacterial infections. *Autophagy*.

[39] Gozuacik D., Kimchi A. (2006). DAPk protein family and cancer. *Autophagy*.

[40] Kim Y. C., Guan K. L. (2015). mTOR: a pharmacologic target for autophagy regulation. *The Journal of Clinical Investigation*.

[41] Bonifazi P., D'Angelo C., Zagarella S. (2010). Intranasally delivered siRNA targeting PI3K/Akt/mTOR inflammatory pathways protects from aspergillosis. *Mucosal Immunology*.

[42] Romani L., Fallarino F., de Luca A. (2008). Defective tryptophan catabolism underlies inflammation in mouse chronic granulomatous disease. *Nature*.

[43] Metz R., Rust S., DuHadaway J. B. (2012). IDO inhibits a tryptophan sufficiency signal that stimulates mTOR: a novel IDO effector pathway targeted by D-1-methyl-tryptophan. *OncoImmunology*.

